# Fe_3_O_4_ nanoparticles as a saturable absorber for giant chirped pulse generation

**DOI:** 10.3762/bjnano.10.107

**Published:** 2019-05-20

**Authors:** Ji-Shu Liu, Xiao-Hui Li, Abdul Qyyum, Yi-Xuan Guo, Tong Chai, Hua Xu, Jie Jiang

**Affiliations:** 1 School of Physics & Information Technology, Shaanxi Normal University, Xi’an 710119, China; 2 Shaanxi Key Laboratory for Advanced Energy Devices, School of Materials Science and Engineering, Shaanxi Normal University, Xi’an 710119, China; 3 Hunan Key Laboratory of Super Microstructure and Ultrafast Process, School of Physics and Electronics, Central South University, Changsha and 410083, China

**Keywords:** erbium laser, Fe_3_O_4_ nanoparticles, fiber lasers, saturable absorber

## Abstract

Fe_3_O_4_ nanoparticles (FONPs) are magnetic materials with a small band gap and have well-demonstrated applications in ultrafast photonics, medical science, magnetic detection, and electronics. Very recently, FONPs were proposed as an ideal candidate for pulse generation in fiber-based oscillators. However, the pulses obtained to date are on the order of microseconds, which is too long for real application in communication. Here, we report the use of FONPs synthesized by a sol–hydrothermal method and used as a saturable absorber (SA) to achieve nanosecond pulses in an erbium-doped fiber laser (EDFL) for the first time. The proposed fiber laser is demonstrated to have a narrow spectral width of around 0.8 nm and a fixed fundamental repetition rate (RPR) of 4.63 MHz, whose spectra and pulse dynamics are different from the mode-locked lasers reported previously. It is demonstrated that the proposed fiber laser based on a FONP SA operates in the giant-chirp mode-locked regime. The most important result is the demonstration of a pulse duration of 55 ns at an output power of 16.2 mW, which is the shortest pulse based on FONPs for EDFLs reported to date. Our results demonstrate that the FONP dispersion allows for an excellent photonic material for application in ultrafast photonics devices, photoconductive detectors, and optical modulators.

## Introduction

Fe_3_O_4_ nanoparticles (FONPs) are excellent magnetic materials, which allows for many applications in various fields such as medical transmission, microwave devices, and optical devices. In addition, FONPs also exhibit nonlinear photonic properties such as two-photon absorption, nonlinear scattering, and optical confinement [1–2]. Ferrous ferric oxide (Fe_3_O_4_) is a transition metal oxide that has a large third-order nonlinear optical susceptibility of χ^(3)^ = 4.0 × 10^−10^ esu and an ultrafast recovery time of 18–30 ps [3]. FONPs can be classified as a semiconductor material (with a band gap of ≈0.3 eV), which can be modulated by tuning the nanoparticle diameter [4]. For the magnetite (Fe_3_O_4_) material of anti-spinel structure, Fe(II) and Fe(III) of the octahedral position of the crystal have been found to produce a charge transfer [5]. Because of the scattering loss, the material absorbs less near infrared (NIR) laser radiation in the NIR-II wavelength range (1000–1300 nm) and the light can penetrate more deeply into the material [6]. In the NIR wavelength region, the optical absorption band does not increase because most of the FONPs have a non-stoichiometric structure [5,7]. Therefore, FONPs are a new and promising nonlinear optical material, which can be widely applied for various photonics applications.

The novel materials can be applied as a saturable absorber (SA) in fiber lasers and can effectively overcome the shortcomings of actively mode-locked lasers, which require an electronic driver and active modulator that make the system more complex and unstable. In 1972, Ippen realized continuous mode-locked pulse output using a dye SA (rhodamine 6G) [8]. Bell labs used an InGaAs/GaAs-on-GaAs superlattice as a SA to realize 1557 nm, 1.2 ps, transformation-limited pulse generation [9]. Following this, carbon nanotubes (CNTs), graphene, topological insulators (TIs), transition metal disulfides (TMDs) and black phosphorus (BP) were used as SAs to realize passively mode-locked lasers [10–39]. Recently, the Bai group used FONPs as a SA to realize Q-switch operation in an erbium-doped fiber laser (EDFL) with a minimum pulse duration of around 3.2 µs [40]. At present, the shortest pulse duration based on FONPs as a saturable absorber is 0.613 µs for fiber lasers [41]. However, to date, only Q-switched fiber lasers based on FONPs with microsecond pulse duration have been reported and no known reports of a FONP-based fiber laser with nanosecond pulse duration are available.

In this work, the shortest pulse has been obtained in a fiber ring laser based on FONPs for the first time. The FONP solution is inserted into a fiber adapter to compose a sandwich structure SA without any polymer, which effectively increases the damage threshold. The modulation depth and the saturation absorption intensity of the FONP SA are 2.5% and 12 MW/cm^2^, respectively. Owing to the superior properties of the prepared FONP SA material, we obtain a laser pulse with a fundamental repetition rate (RPR) of 4.63 MHz and pulse duration of 55 ns at a pump power of 245.2 mW. The proposed pulsed fiber laser based on a FONP SA has a robust structure and good stability and can potentially be used for ultrafast photonics and could play an important role in other nonlinear photonics applications.

## Results and Discussion

**Figure 1:**
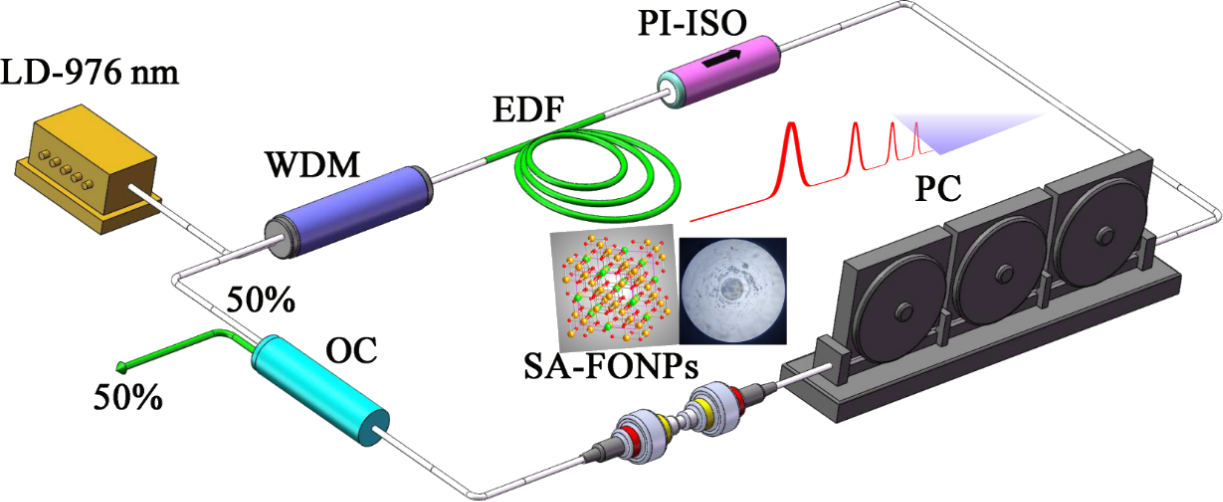
Diagram of the erbium-doped fiber laser with ring cavity and Fe_3_O_4_ nanoparticle saturable absorber.

A schematic of the Er-doped fiber laser based on Fe_3_O_4_ nanoparticles is shown in Figure 1. The fiber laser has a ring cavity of about 43 m in length. In the ring cavity, the pump power of the 980 nm laser diode with a maximum operating power of 510 mW is transmitted into the active fiber using a 980/1550 nm wavelength division multiplexer (WDM). The active medium is supplied by a 0.75 m erbium-doped fiber (EDF) with a 110 dB/m peak absorption coefficient at 1530 nm and a dispersion parameter (D) of −36 ps/nm/km. The EDF has an absorption coefficient of 70 dB/m at 980 nm. The fibers in our cavity are all SMF-28 optical fibers (including the pig-tailed fiber) with a dispersion parameter of 17 ps/nm/km. Therefore, the fiber cavity used in this experiment is dispersion-managed and lies in negative dispersion regime [42]. A polarization independent isolator (PI-ISO) is employed in the cavity to ensure the unidirectional operation of the ring cavity, and a polarization controller (PC) is utilized to adjust the linear cavity birefringence. The solution is connected to a 50/50 optical coupler (OC) with 50% of its output port connected to the combiner. To simultaneously measure two different parameters, a 95.4/4.6 optical coupler is connected to the laser output port. The 4.6% part of the output is connected to a 2 GHz photodetector (Thorlabs DET01CFC), and the detected signal is monitored by a 1 GHz real-time oscilloscope (Rigol DS6104). The 95.4% port is connected to an optical spectrum analyzer (Anritsu MS9710C) with a resolution of 0.05 nm. A radio frequency (RF) spectrum analyzer (Rohde & Schwarz FSC6) is used to measure the signal-to-noise ratio of the pulse.

**Figure 2:**
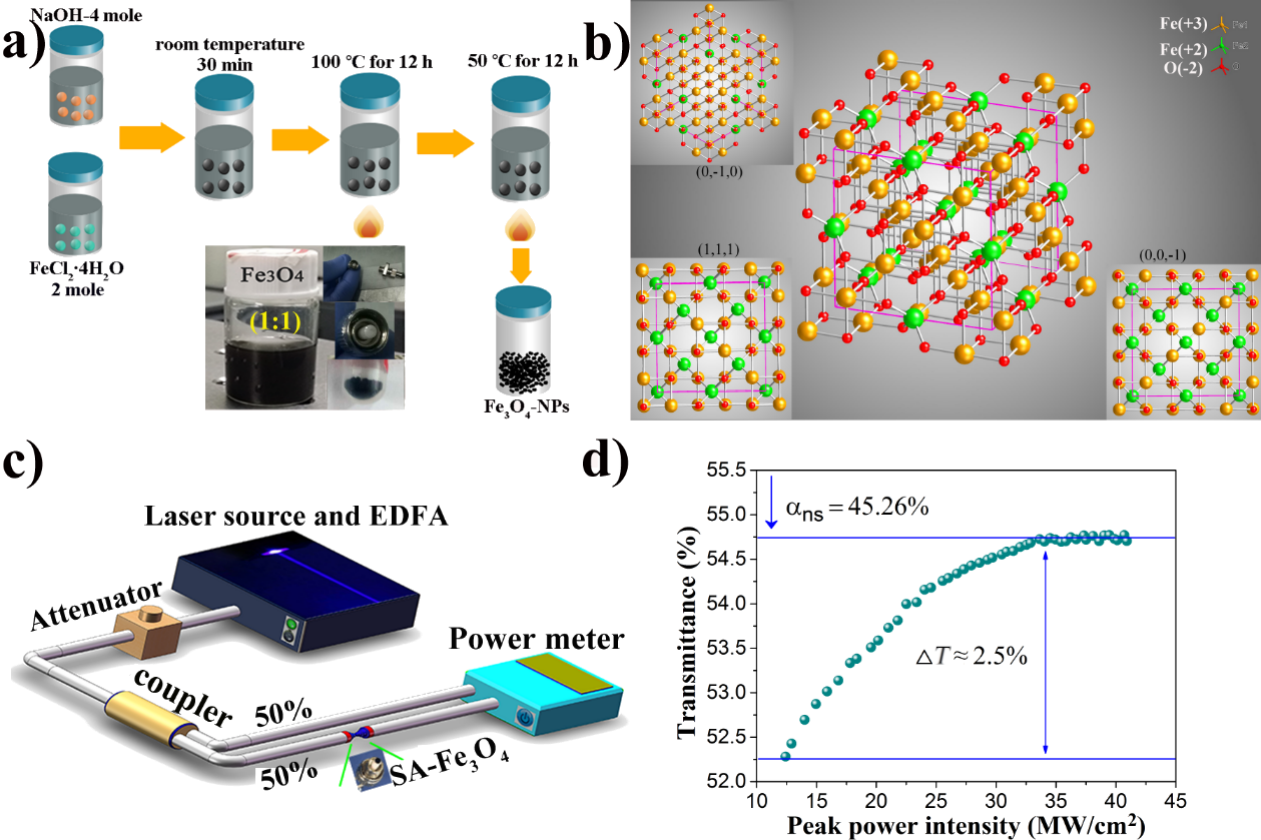
(a) Synthesis process of Fe_3_O_4_ nanoparticles. The inset shows a picture of the sample and the end of the fiber sample. (b) The lattice structure of Fe_3_O_4_. (c) Nonlinear transmission measurement setup for the Fe_3_O_4_ SA. (d) Nonlinear optical absorption characteristics of the FONP-based SA.

There are many methods that can be employed for the synthesis of NPs, such as sol–gel, sol–hydrothermal, co-precipitate, or dry methods. Due to the homogeneity and the versatility of the product, a sol–hydrothermal process was utilized in these experiments. Figure 2a shows the detailed synthesis process of Fe_3_O_4_ nanoparticles. First of all, 20 mL of distilled water and 3.2 g FeCl_2_·4H_2_O are put into a beaker. The beaker then is placed onto a heating/mixing table and stirred at 500 rpm for 30 min. After a half of an hour, a homogeneous solution is obtained. Then 20 mL of distilled water and 4 M sodium hydroxide are put into beaker and mixed for 30 min after which a homogeneous solution is obtained. Next, sodium hydroxide is mixed with FeCl_2_·4H_2_O in a 50 mL Teflon-sealed autoclave and hydrothermal treatment at 100 °C for 12 h is performed. Finally, the resulting product was washed several times with acetone or distilled water and dried in an oven at 50 °C for 12 h to obtain a powder. The insert shows how the FONP powder and acetone were prepared at a 1:1 ratio, and then the FONPs are deposited onto a fiber optic jumper to better interact with the light. The lattice spacing of the Fe_3_O_4_ NP material is shown in Figure 2b. Yellow, green and red circles correspond to positive trivalent iron ions, positive divalent iron ions and negative divalent oxygen ions, respectively. The inserts show three different views of the graph corresponding to the (0,−1,0), (1,1,1), and (0,0,−1) crystal faces. Figure 2c shows the self-designed nonlinear optical measurement system for the FONP SA material. After passing through the coupler, the femtosecond laser light is divided into two channels on average. One path is directly used to measure the output power, and the other is used to measure the output power after passing through the SA. Figure 2d gives the nonlinear transmission curve of the FONP SA. It can be seen that the saturation absorption intensity, modulation depth, and nonsaturable absorbance are about 12 MW/cm^2^, 2.5%, and 45.26%, respectively. These results confirm the applicability of the prepared FONP SA for pulsed fiber lasers.

**Figure 3:**
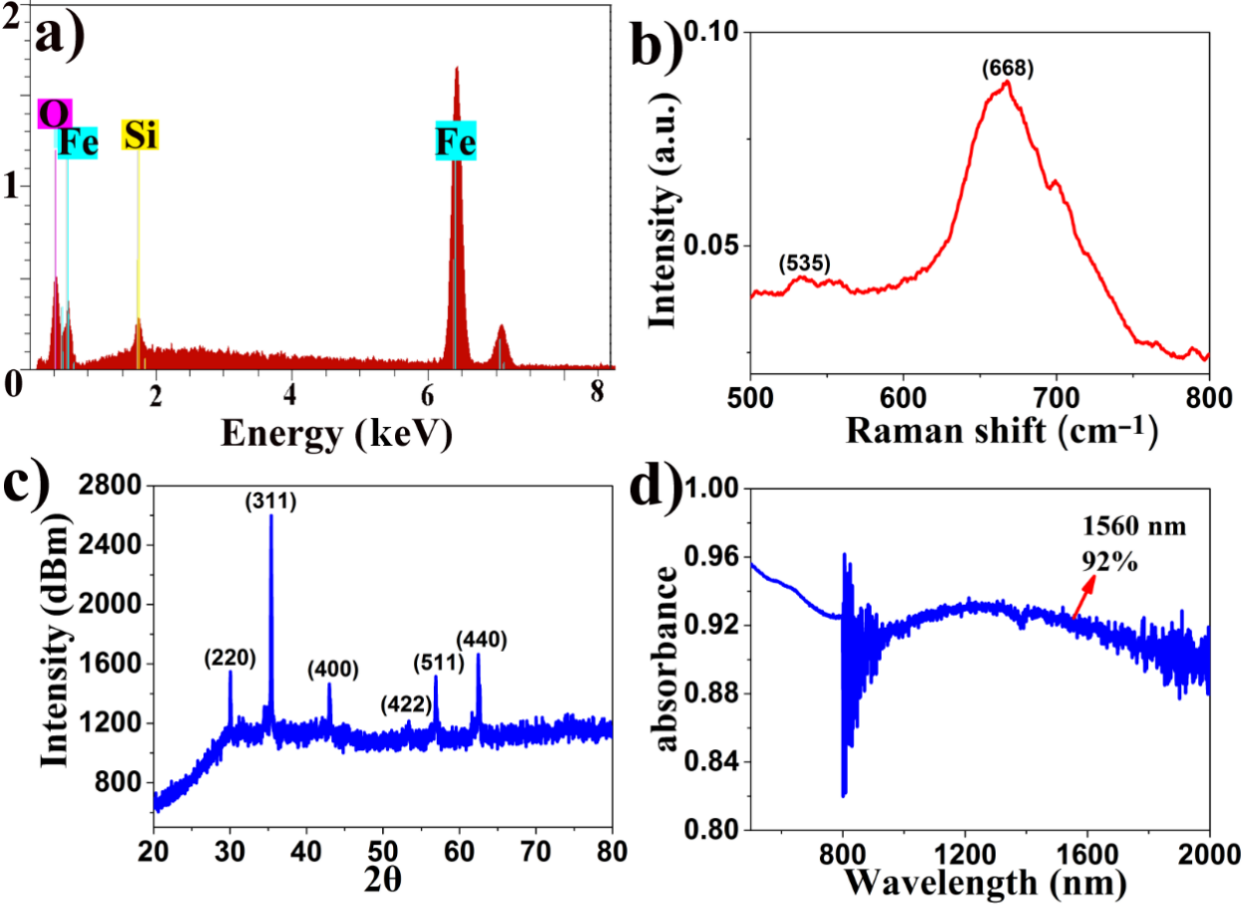
(a) Energy-dispersive spectroscopy, (b) Raman spectroscopy, (c) X-ray diffraction pattern, and (d) UV–vis–NIR spectrum of the as-prepared Fe_3_O_4_ cluster-structured nanoparticles.

The FONP energy-dispersive spectroscopy (EDS) measurement results are shown in Figure 3a. The measured Fe and O elements represent 74.69% and 21.86%, respectively, which are basically consistent with the theoretical value. The 3.45% Si element is associated with the measurement device. The Raman spectra of the as-prepared FONPs were collected using 532 nm laser excitation with an integration time of 15 s, as shown in Figure 3b. The two characteristic peaks of FONPs are located at 535 cm^−1^ and 668 cm^−1^, respectively [7]. Figure 3c shows the crystal diffraction faces of the samples (FONPs) collected with an X-ray diffractometer (XRD) ((220), (311), (400), (422), (511) and (440)), which corresponds well with the JPCDS card number 85-1436 data [7]. The characteristics of the FONP powder were measured by UV–vis–NIR spectroscopy, as shown in Figure 3d. From these results, 92% absorbance at 1560 nm can be observed.

**Figure 4:**
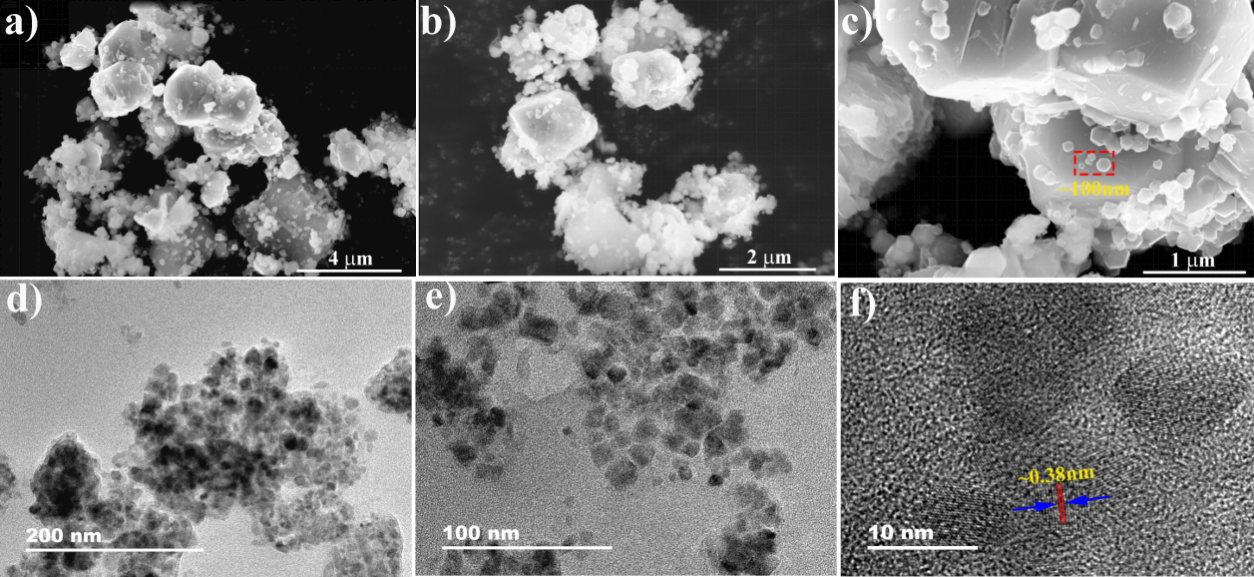
(a, b, c) Scanning electron microscopy, (d, e) transition electron microscopy and (f) high-resolution transmission microscopy images of the FONP saturable absorber.

In Figure 4a–c, the microstructure of the FONP SA is confirmed via scanning electron microscopy (SEM) at the 4, 3 and 1 µm scale, respectively. It can be clearly seen that the diameter of the dispersed nanoparticles is about 100 nm, and the aggregation of the nanoparticles are caused by the magnetic properties of the FNOPs. Figure 4d and 4e show the results observed under a transmission electron microscope (TEM), and the measurement scales are 200 and 100 nm, respectively. Figure 4f shows the results from a high-resolution transmission electron microscope (HR-TEM). Meanwhile, the lattice spacing of the FONP material is found to be about 0.38 nm.

**Figure 5:**
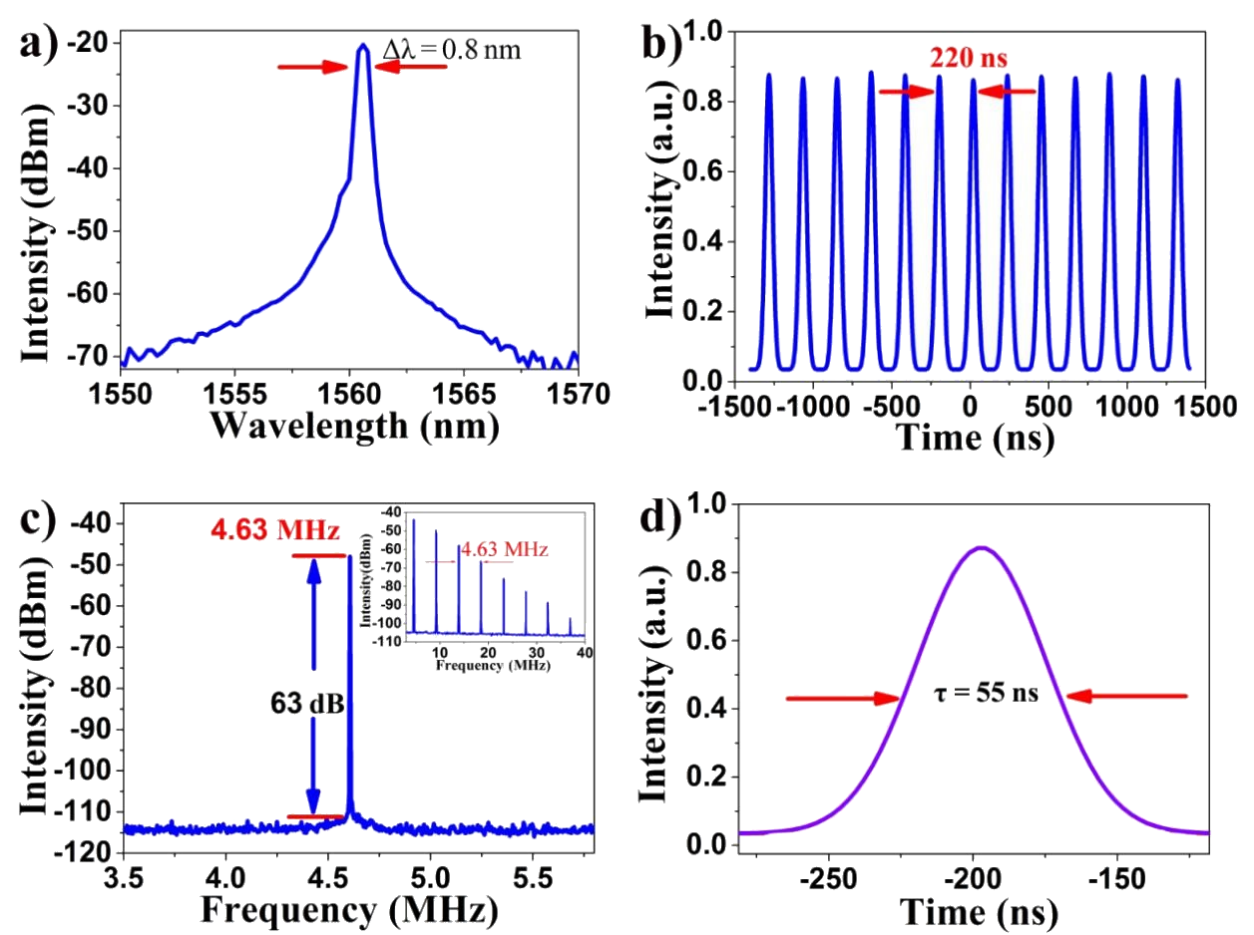
(a) The corresponding spectrum in the fundamental RPR; (b) Oscilloscope trace in the 3 µs range; (c) Radio frequency (RF) spectrum around the fundamental RPR; (d) Single pulse envelope curve with pump power of 245.2 mW.

**Figure 6:**
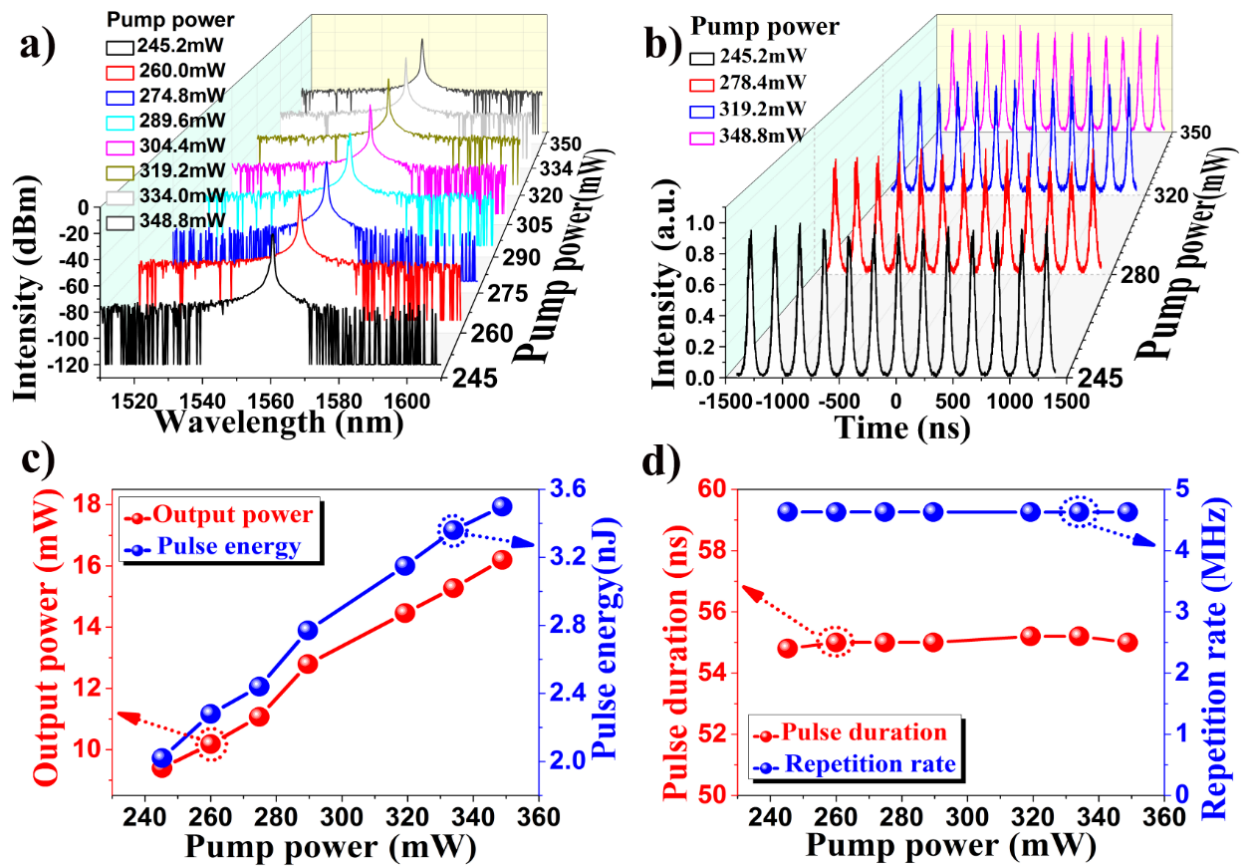
(a) Optical spectrum evolution as a function of pump power, (b) corresponding oscilloscope trace evolution with increasing pump power, (c) output power and pulse energy as a function of pump power, and (d) pulse RPR and pulse duration versus pump power.

The characteristics of the output pulse are summarized in Figure 5 and Figure 6. In Figure 5a, the spectrum shows a smooth shape with a center wavelength of 1560.6 nm and 3 dB bandwidth of 0.8 nm at a pump power of 245.2 mW [41]. As shown in Figure 5b and 5c, the RPR of 4.63 MHz corresponds to a laser cavity length of about 43 m, which is verified by the time interval of the output pulse train. The signal-to-noise ratio (SNR) of 63 dB indicates that the fiber laser operates in a stable state. In Figure 5c, the evolution diagram of SNR intensity with the change of RPR (range about 0–40 MHz) is given, and the repetition interval between them is also 4.63 MHz. The output pulse can be well fit with a Gaussian profile whose full width at half maximum (FWHM) is 55 ns, as shown in Figure 5d.

Figure 6a and 6b show the corresponding spectra and the evolution of the pulses at different pump powers. The spectrum width is maintained near 0.8 nm. The pulse duration of 55 ns and the interval between adjacent pulses of 220 ns are almost unchanged. As the pump power is increased to 245.2 mW, a stable pulse spectrum can be observed. Keeping the polarization state constant, a stable spectrum can be obtained over a wide pump power range from 245.2 mW to 348.8 mW. Figure 6c and 6d summarize the measured average output power and pulse energy, the RPR and pulse duration as a function of the pump power, respectively. By increasing the pump power from 245.2 mW to 348.8 mW, the average output power of the fiber laser based on the Fe_3_O_4_ NP SA grows linearly up to 16.2 mW with a slope efficiency of 5.4% and a maximum output energy of 3.499 nJ. With the increase of pump power, the pulse duration is almost maintained near 55 ns and the fixed fundamental RPR of 4.63 MHz is observed.

To assess the performance of the SA based on FONPs, output of different fiber lasers based on FONPs recently discussed in the literature are summarized in Table 1. We compared several key parameters of the fiber laser, including maximum RPR, maximum output energy, and shortest pulse duration. It can be seen that the maximum RPR is 0.1282 MHz and the shortest pulse duration is 613 ns in current published works. In contrast, we obtain an RPR of 4.63 MHz and pulse duration of 55 ns, which is the shortest pulse duration published to date.

**Table 1:** Comparative output performance of fiber lasers based on Fe_3_O_4_ in recently published works.

Max. RPR (MHz)	Max. energy (nJ)	Min. pulse duration (µs)	Ref.

0.049	≈100	3.5	[43]
0.0734	38.8	3.4	[44]
0.0333	23.76	3.2	[40]
0.0372	322	2.6	[45]
0.1282	321.3	0.613	[41]
4.63	3.499	0.055	this work

It is well-established that the RPR will increase with the increase in pump power when the device is in the Q-switching state [40–41,43–46]. In our results, we demonstrate that as the pump power is increased, the RPR of the fiber laser is fixed at 4.63 MHz as shown in Figure 6d. This result indicates that the output pulse does not operate in the Q-switched regime. In our experiment, the pulse duration of the FONP-based fiber laser is almost around 55 ns without wave-breaking, which is far narrower than that reported in previous works. At the same time, the time bandwidth product of the pulses in the fiber laser is calculated, which exceeds 1000. Thus it can be stated that the laser presented here operates in the mode-locked regime and generates giant-chirp pulses [47–49]. The giant-chirp passively mode-locked fiber laser can be used in many applications, such as chirp pulse amplification, as a seed laser for a high-power fiber laser, which can be potentially applied in certain materials processing, optical coherent detection applications, etc. In addition, in order to check whether some mode-locked pulses are generated in the experiments, we implemented a laser cavity without the SA materials. It was found that only the wavelength can be tuned in the spectra and some fluctuations were observed on the oscilloscope by adjusting the polarization controllers, which is due to the birefringence and nonlinear effect in the fiber laser cavities. It was thus demonstrated that the giant-chirp passive mode locking is mainly caused by the FONP SA material [47–51]. The nonlinear polarization evolution effects observed may influence the operation wavelength and add some noise in the experiments.

## Conclusion

In summary, FONPs prepared via a sol–hydrothermal method were successfully used as a SA to construct a high-performance fiber laser. The surface properties, molecular vibration, structure and composition of the FONPs were systemically studied using SEM, TEM, HR-TEM, EDS, Raman spectra, XRD and UV–vis–NIR. The FONPs exhibited a modulation depth of 2.5%, saturable intensity of 12 MW/cm^2^, and a nonsaturable loss of 45.26%. Employing the FONP SA, we have obtained output pulses with a duration of 55 ns and spectrum bandwidth of 0.8 nm with center wavelength of 1560.6 nm, which have a RPR of 4.63 MHz and output power of 16.2 mW. For the first time, we have obtained giant-chirp passively mode-locked operation in an Er-doped fiber laser based on FONPs as a SA. This work shows that FONPs can not only be used in the application of medicine and magnetism, but also in high-performance, nonlinear optical and ultrafast photonic devices.

## References

[1] Wang H, Pu S, Wang N, Dong S, Huang J (2013). Opt Lett.

[2] Sun G, Dong B, Cao M, Wei B, Hu C (2011). Chem Mater.

[3] Xing G, Jiang J, Ying J Y, Ji W (2010). Opt Express.

[4] El-Diasty F, El-Sayed H M, El-Hosiny F I, Ismail M I M (2009). Curr Opin Solid State Mater Sci.

[5] Tang J, Myers M, Bosnick K A, Brus L E (2003). J Phys Chem B.

[6] Alex A, Povazay B, Hofer B, Popov S, Glittenberg C, Binder S, Drexler W (2010). J Biomed Opt.

[7] Huang C-C, Chang P-Y, Liu C-L, Xu J-P, Wu S-P, Kuo W-C (2015). Nanoscale.

[8] Dienes A, Ippen E, Shank C (1972). IEEE J Quantum Electron.

[9] Zirngibl M, Stulz L W, Stone J, Hugi J, DiGiovanni D, Hansan P B (1991). Electron Lett.

[10] Zhao C, Zhang H, Qi X, Chen Y, Wang Z, Wen S, Tang D (2012). Appl Phys Lett.

[11] Liu M, Zheng X-W, Qi Y-L, Liu H, Luo A-P, Luo Z-C, Xu W-C, Zhao C-J, Zhang H (2014). Opt Express.

[12] Zhang H, Lu S B, Zheng J, Du J, Wen S C, Tang D Y, Loh K P (2014). Opt Express.

[13] Qi Y-L, Liu H, Cui H, Huang Y-Q, Ning Q-Y, Liu M, Luo Z-C, Luo A-P, Xu W-C (2015). Opt Express.

[14] Tu C, Deng Y, Cai M, Huang Z, Li Y, Lu F, Li E (2012). Opt Commun.

[15] Lu S, Zhao C, Zou Y, Chen S, Chen Y, Li Y, Zhang H, Wen S, Tang D (2013). Opt Express.

[16] Shao J, Xie H, Huang H, Li Z, Sun Z, Xu Y, Xiao Q, Yu X-F, Zhao Y, Zhang H (2016). Nat Commun.

[17] Mu H, Lin S, Wang Z, Xiao S, Li P, Chen Y, Zhang H, Bao H, Lau S P, Pan C (2015). Adv Opt Mater.

[18] Liu H, Luo A-P, Wang F-Z, Tang R, Liu M, Luo Z-C, Xu W-C, Zhao C-J, Zhang H (2014). Opt Lett.

[19] Chai T, Li X, Feng T, Guo P, Song Y, Chen Y, Zhang H (2018). Nanoscale.

[20] Wang X-D, Luo Z-C, Liu H, Liu M, Luo A-P, Xu W-C (2014). Appl Phys Lett.

[21] Grelu P, Akhmediev N (2012). Nat Photonics.

[22] Krupa K, Nithyanandan K, Grelu P (2017). Optica.

[23] Sun B, Hu K, Wei Y, Chen D, Gao S, Wang T, He S (2012). Opt Lett.

[24] Zhang X, Wang T, Chen J, Yao H (2018). Opt Lett.

[25] Li X, Dai S, Zou W, Chen J, Nie Q, Dai S (2017). Sci Rep.

[26] Sun Z, Hasan T, Torrisi F, Popa D, Privitera G, Wang F, Bonaccorso F, Basko D M, Ferrari A C (2010). ACS Nano.

[27] Wang F, Rozhin A G, Scardaci V, Sun Z, Hennrich F, White I H, Milne W I, Ferrari A C (2008). Nat Nanotechnol.

[28] Sun Z, Martinez A, Wang F (2016). Nat Photonics.

[29] Li X H, Wang Y S, Zhang W, Zhao W, Hu X H, Yang Z, Gao C X, Wang X L, Liu X L, Shen D Y (2011). Laser Phys.

[30] Li X, Wang Y, Wang Y, Zhao W, Yu X, Sun Z, Cheng X, Yu X, Zhang Y, Wang Q J (2014). Opt Express.

[31] Yu X, Li Y, Hu X, Zhang D, Tao Y, Liu Z, He Y, Haque M A, Liu Z, Wu T (2018). Nat Commun.

[32] Yu X, Yu P, Wu D, Singh B, Zeng Q, Lin H, Zhou W, Lin J, Suenaga K, Liu Z (2018). Nat Commun.

[33] Yan P, Liu A, Chen Y, Wang J, Ruan S, Chen H, Ding J (2015). Sci Rep.

[34] Nyushkov B N, Denisov V I, Kobtsev S M, Pivtsov V S, Kolyada N A, Ivanenko A V, Turitsyn S K (2010). Laser Phys Lett.

[35] Chernysheva M, Rozhin A, Fedotov Y, Mou C, Arif R, Kobtsev S M, Dianov E M, Turitsyn S K (2017). Nanophotonics.

[36] Tao Y, Yu X, Li J, Liang H, Zhang Y, Huang W, Wang Q J (2018). Nanoscale.

[37] Yu X, Dong Z, Liu Y, Liu T, Tao J, Zeng Y, Yang J K W, Wang Q J (2016). Nanoscale.

[38] Turitsyn S K, Bednyakova A E, Fedoruk M P, Papernyi S B, Clements W R L (2015). Nat Photonics.

[39] Churkin D V, Sugavanam S, Vatnik I D, Wang Z, Podivilov E V, Babin S A, Rao Y, Turitsyn S K (2015). Adv Opt Photonics.

[40] Bai X, Mou C, Xu L, Wang S, Pu S, Zeng X (2016). Appl Phys Express.

[41] Chen Y, Yin J, Chen H, Wang J, Yan P, Ruan S (2017). IEEE Photonics J.

[42] Song Y F, Zhang H, Zhao L M, Shen D Y, Tang D Y (2016). Opt Express.

[43] Mao D, Cui X, Zhang W, Li M, Feng T, Du B, Lu H, Zhao J (2017). Photonics Res.

[44] Al-hayali S K M, Al-janabi A H (2018). Laser Phys.

[45] Koo J, Lee J, Kim J, Lee J H (2018). J Lumin.

[46] Li J, Luo H, Bo Z, Lu R, Guo Z, Han Z, Liu Y (2016). Sci Rep.

[47] Renninger W H, Chong A, Wise F W (2008). Opt Lett.

[48] Wang L R, Liu X M, Gong Y K (2010). Laser Phys Lett.

[49] Shi M W, Liu B W, Wang S J, Chai L, Wang C Y (2012). Chin J Lasers (Chin Ed).

[50] Lu H, Zhou P, Wang X, Jiang Z (2015). IEEE Photonics J.

[51] Churkin D V, Sugavanam S, Tarasov N, Khorev S, Smirnov S V, Kobtsev S M, Turitsyn S K (2015). Nat Commun.

